# Prevalence and associated risk factors of self-reported symptoms suggestive of female genital schistosomiasis (FGS) among women in KwaZulu-Natal, South Africa

**DOI:** 10.1371/journal.pntd.0014633

**Published:** 2026-08-26

**Authors:** Sinegugu Shongwe, Chester Kalinda, Khumbulani Hlongwana, Themba Ginindza

**Affiliations:** 1 College of Health Sciences, Discipline of Public Health, School of Medicine, University of KwaZulu-Natal, Durban, South Africa; 2 School of Global Health, University of Global Health Equity (UGHE), Kigali, Rwanda; 3 Cancer & Infectious Diseases Epidemiology Research Unit (CIDERU), Discipline of Public Health, School of Medicine, University of KwaZulu-Natal, Durban, KZN, SA; Huazhong University of Science and Technology Tongji Medical College, CHINA

## Abstract

**Background:**

Female genital schistosomiasis (FGS) is a neglected tropical disease affecting women and girls in schistosomiasis-endemic areas. However, it remains under-recognised because of limited access to FGS-appropriate diagnostic approaches. Symptom-based reporting is often used to estimate the burden of FGS. This study aimed to determine the prevalence and identify the associated risk factors of self-reported FGS-suggestive symptoms among women in KwaZulu-Natal, South Africa.

**Methods:**

A cross-sectional study was conducted from June to August 2025 using a structured, interviewer-administered questionnaire. 713 women were enrolled, of whom 695 provided complete data and were included in the analysis. Data were processed and analysed using Stata 19.5 SE. Descriptive statistics were used to summarise the data. The Pearson chi-square test was used to assess the association between sociodemographic characteristics and symptoms suggestive of FGS, and a multivariable logistic regression model was used to identify the independent predictors of these symptoms. Wald tests were used to assess the overall significance of the predictors, and model fit was evaluated using the Hosmer–Lemeshow test.

**Results:**

Overall, 45.9% (95%CI: 42.2–49.6) reported experiencing ≥1 suggestive FGS symptom, and this differed across age groups, with the highest proportion observed in the 30–39 years age group (53.2%; 95% CI: 46.6–59.8). Lower abdominal pain (n = 130; 40.8%) and vaginal itching (n = 110; 34.5%) were the most common symptoms. In the overall model, religious affiliation (*p* = *0.001*), income source (*p < 0.001*), and awareness of FGS (*p < 0.001*) were statistically significantly associated with reporting symptoms suggestive of FGS.

**Conclusion:**

Nearly half of the women in this schistosomiasis-endemic setting reported FGS-suggestive symptoms, providing important evidence for incorporating symptom screening into existing reproductive and community health worker guidelines to strengthen the early detection and referral of women at risk of FGS.

## Author summary

Female genital schistosomiasis (FGS) is a neglected disease caused by the parasite Schistosoma haematobium. An estimated 56 million women and girls in sub-Saharan Africa are affected, but the disease is poorly recognised. Symptoms such as lower abdominal pain, vaginal itching, abnormal vaginal discharge, and blood in the urine are often mistaken for sexually transmitted infections or other gynaecological conditions, leading to diagnosis and treatment delays. We surveyed 713 women of reproductive age who presented to primary healthcare clinics in the iLembe District of KwaZulu-Natal, South Africa, an area of ongoing schistosomiasis transmission. However, 695 women had complete data and were included in this analysis. Although almost half (45.9%) of the participants reported at least one symptom suggestive of FGS, only 15.4% reported prior knowledge of the disease. Women who identified with Traditional African Religion, relied on remittances for income, and were aware of FGS, were more likely to report symptoms. Our findings demonstrate the high burden of symptoms potentially attributable to FGS in the study setting. Incorporating FGS screening into routine reproductive health services, improving healthcare worker training, and increasing community education may improve the early identification and management of FGS.

## Introduction

Female Genital Schistosomiasis (FGS) is a gynaecological manifestation of *S. haematobium* infection that arises when parasite eggs are deposited in the female genital tract, predominantly in the cervix, vagina, and vulva [1–3]. It is estimated that at least 56 million women and girls in sub-Saharan Africa (SSA) are affected, making it one of the most prevalent yet least recognised gynaecological conditions on the continent [1,4]. In endemic areas of SSA, the prevalence of FGS ranges from 10.5% to 75% [5,6]. Despite its high burden, the disease is often misdiagnosed as a sexually transmitted infection (STI), cervical pathology, or urinary tract infection (UTI) because of overlapping clinical presentations, including blood in urine, abnormal vaginal discharge, lower abdominal pain, post-coital bleeding, pain during sex, and vaginal itching [3,6,7]. This diagnostic confusion has significant consequences, including unnecessary antibiotic treatment, stigmatisation, and missed opportunities for targeted anti-schistosomal therapy [8,9]. The clinical and reproductive health consequences of FGS are substantial [10,11].

These outcomes disproportionately affect young women of reproductive age in rural, resource-limited settings, where schistosomiasis transmission is active and access to specialist care is constrained [12]. Awareness of FGS at the community and health system levels in endemic settings is notably limited [13]. Healthcare workers in sub-Saharan Africa frequently lack training in the identification and clinical management of FGS, and affected women may not associate gynaecological symptoms with waterborne parasitic exposure [11,14]. This awareness gap impedes early presentation for care, reinforces diagnostic delays, and perpetuates the cycle of unrecognised morbidity [15].

South Africa remains endemic for *S. haematobium*, with more than 4 million people estimated to be infected with schistosomiasis, particularly in the subtropical and rural regions of KwaZulu-Natal (KZN), Mpumalanga, and Limpopo provinces [16]. Approximately 5 million South Africans living in poor rural areas lack access to clean water [17]. In rural areas without piped water, communities near water bodies (rivers, ponds, lakes, and dams) are often infected with schistosomiasis [18]. In KZN, environmental conditions such as freshwater bodies harbouring *Bulinus* spp. snail intermediate hosts, combined with persistent water-contact activities for domestic use, sustain transmission [16,19]. The iLembe District Municipality in KZN has been identified as a historically high-burden area, with prior surveys documenting *S. haematobium* infection among school children and reported water-contact behaviours in communities proximate to freshwater sources [20]. However, population-level data on FGS-suggestive symptoms and their determinants among adult women of reproductive age attending primary healthcare facilities in this district remain limited.

To address population-level data on the sparsity of FGS-suggestive symptoms and provide much-needed epidemiological evidence, this study aimed to estimate the prevalence of self-reported symptoms suggestive of FGS and identify factors associated with symptom reporting among women of reproductive age attending primary healthcare clinics in Ndwedwe and Mandeni municipalities in the iLembe District, KZN, South Africa. The findings are intended to inform targeted public health responses and contribute to the broader evidence base supporting the integration of FGS management into primary health care services in endemic settings.

## Methods and materials

### Ethical statement

Ethical approval was obtained from the University of KwaZulu-Natal Biomedical Research Ethics Committee (***Ref. no. BREC/00008448/2025***), the KwaZulu-Natal Provincial Health Research Ethics Committee (***Ref.no. KZ_202502_023***), and gatekeeper permission from the iLembe District Health Office and PHCs’ operational managers. Participation was voluntary, written informed consent was obtained from all participants, and confidentiality was ensured using unique study identification numbers. Participants were informed of the study procedures, risks, and benefits, and their right to withdraw at any time without affecting access to healthcare. All data were handled confidentially and used only for research purposes.

### Study area and setting

This study was conducted in the iLembe District, KZN, South Africa, in two local municipalities, namely: Ndwedwe and Mandeni, which form part of the district’s four local municipalities. Both Ndwedwe and Mandeni are classified as rural. These municipalities were selected as implementation sites for the parent project, Dual Screening by Spectral Artificial Visual Examination (SAVE) for Female Genital Schistosomiasis (FGS) and Cervical Cancer (acronymed as DUAL-SAVE-FGS), a clinical research initiative aimed at improving the detection and management of FGS. The selection was informed by existing evidence documenting the presence of Schistosoma haematobium intermediate host snails (Bulinus spp.) and reported water-contact activities within communities, indicating historical exposure risk in these areas [20]. Within these municipalities, eight primary healthcare clinics (four per municipality) were purposively selected as study sites based on two criteria: their location within the DUALSAVE-FGS project implementation areas and their proximity to communities and schools previously identified as high-burden areas through prior mapping of S. haematobium intermediate host snails and water-contact behaviours in the iLembe District [20]. The total number of PHC clinics across Ndwedwe and Mandeni municipalities was not formally enumerated in this study. The purposive selection of four clinics per municipality was guided by documented transmission risk and established community access rather than administrative convenience (S1 Table).

### Study design and population

A facility-based analytic cross-sectional study was conducted between June and August of 2025. The target population comprised women aged 18–49 years who attended eight selected primary healthcare clinics for routine health services during the study period. Although the WHO defines the reproductive age group as 15–49 years, this study enrolled women aged 18–49 years, as South African law classifies individuals under 18 years as minors requiring additional parental or guardian consent procedures, which were beyond the operational scope of this facility-based study. Participants were eligible if they resided in communities served by the participating primary healthcare clinics. Clinic catchment areas were defined using the service boundaries assigned to each facility by the iLembe District Health Office, which delineates the populations routinely accessing care at those clinics. Women who provided written informed consent and met the inclusion criteria were eligible for participation.

### Sample size

The sample size was determined based on the previously reported prevalence of schistosomiasis in the study area [21]. Calculations were performed at a 95% confidence level using the formula proposed by Lwanga et al. [22] for estimating a population proportion with specified relative precision: $n=\frac{Z^{\text{2}}(\text{1}-p)}{\varepsilon^{\text{2}}p}$

where n is the required sample size, p is the estimated prevalence, Z is the standard normal deviate corresponding to a 95% confidence level, and ε is the relative precision. A reported prevalence of 32% was used for sample size calculation. This estimate was derived from previous analyses of baseline genital symptom data among schoolgirls and young women in rural KwaZulu-Natal, which suggested that approximately 32% of female learners may present with genital symptoms not attributable to sexually transmitted infections and potentially associated with schistosomiasis [23]. This estimate was considered an appropriate proxy for FGS risk in schistosomiasis-endemic settings within the same province. With a relative precision of 5%-10% of the true prevalence (0.05 < ε < 0.10), a minimum sample size of 713 participants was deemed adequate for the study.

### Sampling

A probability sampling approach was employed using stratified sampling, with municipalities (Ndwedwe and Mandeni) serving as the primary strata to ensure representation from both parent project implementation sites. The sampling was conducted in two stages.

In the first stage, probability-proportional-to-size (PPS) sampling was used to allocate the total target sample size (n = 713) across the eight selected primary healthcare clinics (S1 Table). Clinic-level administrative data were obtained from the KwaZulu-Natal Provincial Department of Health’s District Health Information System (DHIS). The calculated sample sizes allocated to each clinic are shown in S1 Table. For example, the Isithebe Clinic accounted for 40.55% of the total annual outpatient headcount (84,894 of 209,370); therefore, 289 participants were allocated to this clinic (40.55% × 713). Clinic-level headcount data for June–August 2024, confirming the operational feasibility of the PPS-allocated recruitment targets at each of the eight study sites, are presented in S2 Table.

In the second stage, consecutive enrolment was conducted at each clinic. All women attending the selected primary healthcare (PHC) clinics during the data collection period were screened for eligibility. Women aged 18–49 years who resided within the clinic’s catchment area and provided written informed consent were consecutively enrolled until the clinic-specific sample size was reached. Women who were too ill to participate, unwilling, or unable to provide informed consent were excluded, and recruitment proceeded to the next eligible potential participant.

### Data collection

Data were collected by trained enumerators throughout the week from women of reproductive age attending routine care at the clinic, using a structured, interviewer-administered questionnaire. The questionnaire was implemented using Research Electronic Data Capture (REDCap) [24] software on Android tablets. The questionnaire comprised structured, closed-ended questions and took approximately 20–30 minutes to complete. It was organised into multiple sections addressing different components of the study. As participants were already present at the clinics for routine services, travel reimbursements were not provided; however, airtime vouchers were offered at the end of the interview as a token of appreciation for their time.

### Operationalisation of variables

Socio-demographic and exposure variables were collected and operationalised as follows. Age was categorised into 18–29, 30–39, and 40–49 years groups. Residence was classified as urban or rural, and marital status was dichotomised as single (never married) or in a union. Religious affiliations included Christianity, Traditional African Religion (encompassing the Nazareth Baptist Church and ancestral customs), and the other category, Islam, Hinduism, and other non-Christian, non-African traditional religions. Socioeconomic variables comprised primary income source (formal employment, business/farming, government grant, remittance). These categories reflect the primary livelihood typologies in rural KwaZulu-Natal and represent distinct socioeconomic strata with different implications for water, sanitation and hygiene (WASH) access and healthcare utilisation, educational attainment (none, primary, secondary/high, tertiary), and monthly income categories (≤$55, $55–$274,95, ≥ $275, or prefer not to say) were converted from South African Rand (ZAR) to United States Dollars (USD) using the South African Reserve Bank exchange rate [applicable on June 2025]. Exposure-related variables included self-reported access to clean water (no, yes, sometimes) and primary water-contact activity (bathing or other; the latter a composite of washing, household chores, and cooking). Bathing was classified separately, given that it represents the most sustained and whole-body form of freshwater contact and carries the highest potential for cercarial exposure. These two variables were measured as distinct constructs, reflecting the fact that access to improved water infrastructure does not preclude ongoing contact with natural freshwater bodies for domestic, recreational, or cultural purposes. Freshwater exposure was further characterised through participants’ identification of the nearest river or dam, whether they used a natural water source as their primary water source, and which specific activities they performed in natural water bodies, including bathing, washing clothes, swimming, and other uses. A composite variable capturing any freshwater contact activity was derived from these responses. Finally, awareness of Female Genital Schistosomiasis (FGS) was recorded as yes/no, with sources of information (healthcare workers, family/friends, traditional healer, media) reported by those aware.

### Self-reported symptoms suggestive of Female Genital Schistosomiasis (FGS)

In settings where diagnostic tools for FGS are limited or unavailable, symptom-based reporting is often used as a pragmatic proxy to estimate the FGS burden in population studies. Participants were asked whether they had ever experienced any of the following symptoms commonly associated with FGS: blood in the urine, lower abdominal pain, pain during sexual intercourse, bleeding after sexual intercourse, vaginal itching, or abnormal vaginal discharge. Participants were permitted to report more than one symptom, and a “none” option was included.

For analytical purposes, a binary outcome variable was derived to estimate the prevalence of symptoms suggestive of FGS in the study population. Participants who reported at least one symptom were classified as having FGS-suggestive symptoms. In contrast, those who did not report any symptoms were classified as having no FGS-suggestive symptoms. These symptoms were used as self-reported indicators suggestive of FGS rather than as clinical confirmation of the condition. This approach enabled the estimation of the prevalence of FGS-suggestive symptoms and associated risk factors among adult women in KwaZulu-Natal, a population with limited epidemiological data on FGS.

### Data analysis

All analyses were conducted using Stata/SE version 19.5 (StataCorp, College Station, Texas, USA). The data were cleaned and checked for errors and missing values. Eighteen records were excluded from the final analytical dataset because of incomplete information. Descriptive statistics were used to summarise the sociodemographic characteristics, with continuous variables summarised using the mean and standard deviation, and categorical variables summarised using frequencies and percentages. The prevalence of self-reported symptoms suggestive of FGS was reported as a proportion with 95% confidence intervals (CIs). The association between the sociodemographic and exposure variables was explored using Pearson’s chi-square test or Fisher’s exact test when the expected cell count was less than 5. Variables with a p-value <0.20 in the bivariate analyses were selected for inclusion in the initial multivariable logistic regression model. A more liberal threshold was used to avoid premature exclusion of potentially important variables that may become significant after adjusting for other covariates. This approach is commonly recommended in epidemiological modelling to improve confounder identification and model stability [25,26]. Backwards selection multivariable logistic regression was used to identify factors independently associated with the outcome, while controlling for other variables. The strength and significance of individual associations in the multivariable model were assessed using robust Wald tests and reported as adjusted odds ratios (aORs) with 95% CI.

## Results

### Characteristics of the study population

The mean age of the participants was 30.0 ± 8.3 years, with 52.5% (n = 364) aged 18–29 years. The majority of participants (91.8%, n = 638) resided in rural areas and were single/ unmarried (96.5%, n = 671). Most participants identified as Christians (88.2%, n = 613), and over two-thirds were unemployed (68.4%, n = 475). Approximately one-third (30.1%, n = 209 respondents) reported government grants as their primary source of income. More than half of the participants (58.3%, n = 405) had completed secondary or high school education, and 43.2% (n = 300) reported a monthly income of ≤$55. Most participants reported access to clean water (81.9%, n = 569), and bathing was the most common water-contact activity (86.9%, n = 604). Awareness of female genital schistosomiasis was low (15.4%, n = 107). Among all participants, the primary sources of general health information were the media (65.6%, n = 456) and healthcare workers (31.5%, n = 219), with minimal input from family/friends or traditional healers (each 1.4%, n = 10) (Table 1).

**Table 1:** Socio-demographic characteristics of the study population (N = 695).

Demographics	n (%)
Age: n (%)	
Mean ± SD Range	30.02 ± 8.28
18-29 years	364 (52.45)
30-39 years	216 (31.12)
40-49 years	114 (16.43)
Residence: n (%)	
Rural	638 (91.80)
Urban	57 (8.20)
Marital status: n (%)	
Single (Never married)	669 (96.54)
Married/in Union	24 (3.46)
Religion: n (%)	
Christianity	613 (88.20)
Traditional African Religion	32 (4.60)
Others *	50 (7.19)
Income source: n (%)	
Employer	203 (29.21)
Business/Farming	17 (2.45)
Government	209 (30.07)
Remittance	266 (38.27)
Education: n (%)	
Never been to school	7 (1.01)
Primary	201 (28.92)
Secondary/High	405 (58.27)
Tertiary	82 (11.80)
Monthly Wage: n (%)	
≤$55	300 (43.23)
$55–$274,95	294 (42.36)
≥$275	34 (4.90)
Prefer not to say	66 (9.51)
^¥^Access to clean water: n (%)	
None	97 (13.96)
Yes	569 (81.87)
Sometimes	29 (4.17)
^¥^Water contact activities: n (%)	
Bathing	604 (86.91)
Others^#^	91 (13.09)
FGS awareness: n (%)	
No	588 (84.60)
Yes	107 (15.40)
^+^Source of general health information: n (%)	
Healthcare workers	219 (31.51)
Family/friends	10 (1.44)
Traditional healer	10 (1.44)
Media	456 (65.61)

*“Other” religion includes religious affiliations such as Islam, Hinduism, and other non-Christian, non-Traditional African spiritual beliefs.

# “Others” Water contact activities include washing clothes, drinking, swimming, cooking and farming.

+ The source of general health information was assessed among all participants (N = 695) and reflects the channels through which participants generally access health information, not FGS-specific information.

¥Access to clean running water and water contact activities reflect distinct constructs measured separately in the questionnaire. Access to clean running water refers to the availability of improved water infrastructure. In contrast, water contact activities capture behaviours performed in natural freshwater sources such as rivers and dams, irrespective of household water supply.

### Overall prevalence of symptoms suggestive of FGS

Overall, 45.9% (319/695; 95%CI: 42.2–49.6) reported at least one symptom suggestive of FGS. Prevalence differed across age groups, with the highest proportion observed among women aged 30–39 years (53.2%; 95%CI: 46.6–59.8), compared to those aged 18–29 years (41.5%; 95%CI: 36.5–46.6) and 40–49 years (45.6%; 95%CI: 36.7–54.8). The difference in prevalence across age groups was statistically significant (*p = 0.023*). Symptom reporting differed significantly by religion (*p < 0.001*), with a substantially higher proportion observed among those identifying with the Traditional African religion (84.4%; 95%CI: 67.5–93.4) compared with those identifying as Christian (44.7%; 95%CI: 40.8–48.7). Water-contact activities were associated with reported symptoms *(p = 0.048*). Participants who reported bathing as their primary water-contact activity had a higher prevalence of symptoms (47.4%; 95%CI: 43.4–51.3) than those who reported other activities (36.3%; 95%CI: 27.1–46.6). Awareness of FGS was also strongly associated with symptom reporting, with a higher prevalence among those who reported prior awareness (64.5%; 95%CI: 55.0–73.0) compared to those who were unaware (42.5%; 95%CI: 38.6–46.6) (*p < 0.001*). No statistically significant associations were observed between education level (*p = 0.499*), place of residence (*p = 0.431*), monthly income (*p = 0.852*), and access to clean water (*p = 0.679*) and the outcome variable in the bivariate analyses (Table 2).

**Table 2:** Overall prevalence by socio-demographic characteristics (N-695).

Characteristic	Proportion (95%CI)	p-value*
**Overall prevalence**	**45.9 (42.15-49.69)**	
**Age group (years)**		0.023
18-29 years	41.5 (36.5 – 46.6)	
30-39 years	53.2 (46.6 –59.8)	
40-49 years	45.6 (36.7 –54.8)	
**Education**		0.499
None	42.9 (14.3 – 77.1)	
Primary	50.3 (43.4 – 57.1)	
Secondary/High	43.7 (38.9 – 48.6)	
Tertiary	46.3 (35.9 – 57.2)	
**Residence**		0.431
Rural	45.5 (41.6 – 49.3)	
Urban	50.9 (38.1 –63.5)	
**Marital status**		0.683
Single (Never married)	45.9 (42.1 – 49.7)	
Married/in Union	41.7 (24.1 – 61.7)	
**Religion**		0.000
Christianity	44.7 (40.8 – 48.7)	
Traditional African Religion	84.4 (67.5 –93.4)	
Others ^#^	36.0 (24.0 –50.1)	
**Income source**		0.006
Employer	51.2 (44.4 –58.1)	
Business/Farming	64.7 (40.4 – 83.2)	
Government	49.2 (42.5 – 56.0)	
Remittance	38.0 (32.3 – 44.0)	
**Monthly wage**		0.852
≤$55	46.0 (40.4 – 51.7)	
$55–$274,95	46.9 (41.3 – 52.7)	
≥$275	47.1 (31.2 – 63.6)	
Prefer not to say	40.9 (29.7 – 53.1)	
**Access to clean water**		0.679
None	48.5 (38.7 – 58.3)	
Yes	45.2 (41.1 – 49.3)	
Sometimes	51.7 (34.1 – 69.0)	
**Water contact activities**		0.048
Bathing	47.4 (43.4 – 51.3)	
Others	36.3 (27.1 – 46.6)	
**FGS Awareness**		0.000
No	42.5 (38.6 – 46.6)	
Yes	64.5 (55.0 – 73.0)	
**Source of health information**		0.647
Healthcare workers	45.7 (39.2 – 52.3)	
Family/friends	60.0 (29.7 – 84.2)	
Traditional healer	60.0 (29.7 – 84.2)	
Media	45.4 (40.9 –50.0)	

*p-values were derived from chi-square tests comparing proportions across categories.

# Other” religion includes religious affiliations such as Islam, Hinduism, and other non-Christian, non-Traditional African spiritual beliefs^.^

### Distribution of self-reported symptoms suggestive of female genital schistosomiasis among women

**Fig 1:**
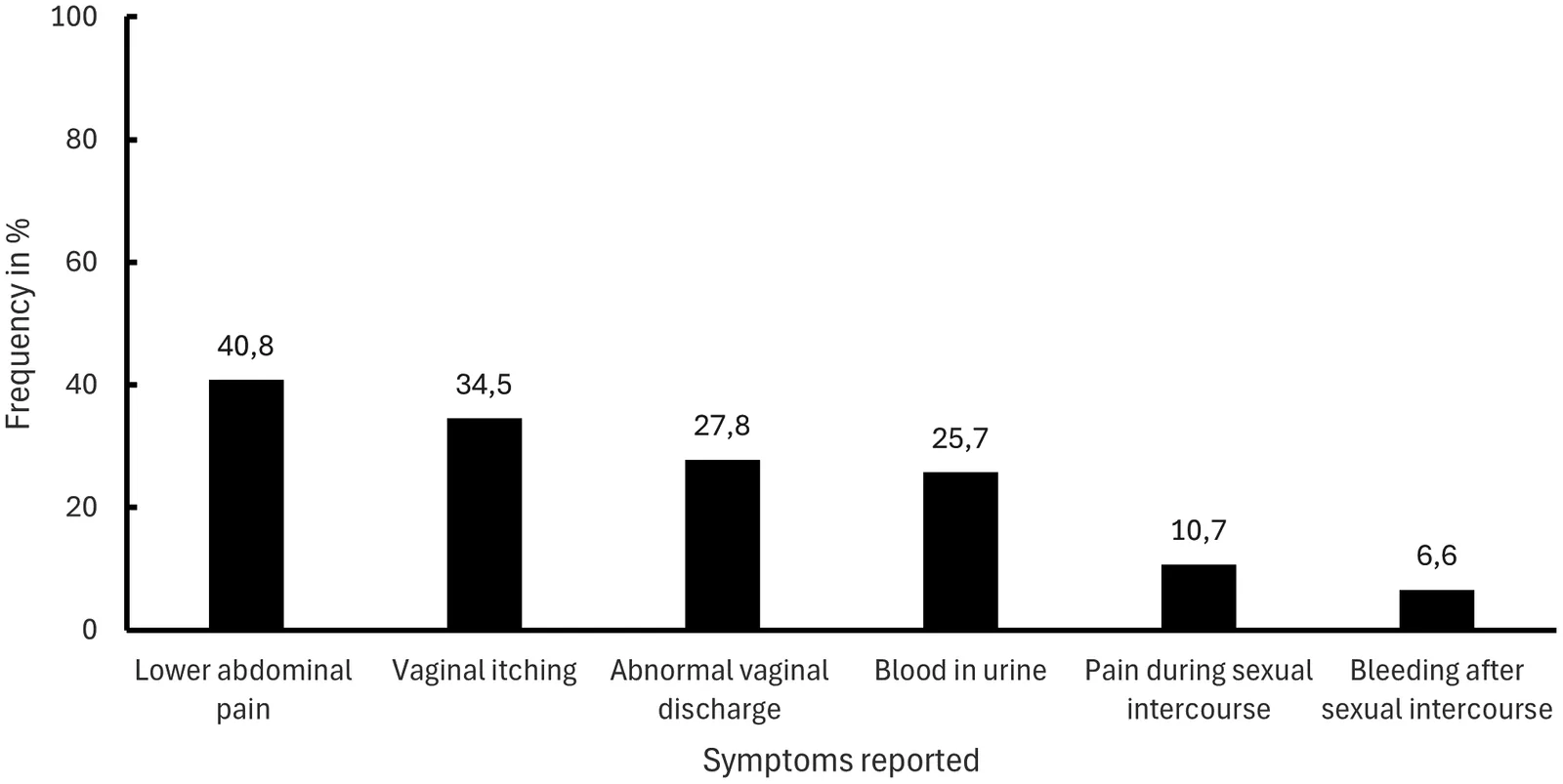
Distribution of self-reported symptoms suggestive of female genital schistosomiasis among women (N = 695).

A total of 319 (45.9%) women reported at least one FGS-suggestive symptom, with lower abdominal pain, vaginal itching, abnormal vaginal discharge, and blood in the urine being the most commonly reported (40.8%, 34.5%, 27.8%, and 25.7%, respectively). In comparison, symptoms included pain during sexual intercourse (10.7%) and bleeding after sexual intercourse (6.6%) were less reported (Fig 1). The distribution of symptom combinations among symptomatic women, including the frequency of single- and multiple-symptom reports, is presented in S3 Table.

### Risk factors associated with self-reported symptoms related to FGS

Among the sociodemographic characteristics used in the overall model, religious affiliation (*p = 0.001*), income source (*p = < 0.001*), and Awareness of FGS (*p = < 0.001*) were significantly associated with reporting symptoms suggestive of FGS. Age (*p = 0.112*) and water-contact activity (*p = 0.050*) were not statistically significant variables. Women identifying with African traditional religions were 6.37-fold (aOR = 6.37; 95%CI: 2.38–17.04; *p =<0.001*) more likely to report symptoms than Christian participants. Women whose primary source of income was employment were 51% (aOR = 1.51; 95% CI: 0.50–4.54; *p = 0.465*) more likely to report symptoms associated with FGS than women who relied on remittances, although this association was not statistically significant. Participants who were aware of FGS were 2.99-fold (aOR = 2.99; 95%CI: 1.89–4.71; *p = < 0.001*) more likely to report symptoms than those who were unaware. Women who engaged in water-contact activities other than bathing were 33% (aOR = 0.67; 95%CI: 0.41–1.08; *p = 0.051*) less likely to report symptoms than those who bathed in freshwater (Table 3).

**Table 3:** Risk factors associated with self-reported symptoms suggestive of female genital schistosomiasis (N = 695).

Variable	Un-OR^i^ (95% CI)	p-value	AOR^ii^ (95% CI)	p-value	AWT^iv^ p-value
**Age group (years)**					0.112
18–29	Ref	—	Ref	—	
30–39	1.07 (0.86–1.32)	0.541	1.38 (0.96–1.97)	0.084	
40–49	1.07 (0.86–1.32)	0.541	0.90 (0.57–1.43)	0.662	
**Religion**					0.001
Christianity	Ref	—	Ref	—	
Traditional African religion	1.16 (0.87–1.53)	0.312	**6.37 (2.38–17.04)**	**<0.001**	
Other	1.16 (0.87–1.53)	0.312	0.76 (0.40–1.43)	0.396	
**Income source**					<0.001
Employer	Ref	—	Ref	—	
Business/Farming	0.84 (0.73–0.95)	0.007	1.51 (0.50–4.54)	0.465	
Government	0.84 (0.73–0.95)	0.007	1.27 (0.83–1.93)	0.273	
Remittance	0.84 (0.73–0.95)	0.007	**0.61 (0.37–0.85)**	**0.017**	
**Water-contact activity**					0.050
Bathing	Ref	—	Ref	—	
Other activities	0.67 (0.41–1.08)	0.100	0.67 (0.41–1.08)	0.105	
**FGS awareness**				<0.001	
No	Ref	—	Ref	—	
Yes	**2.50 (1.62–3.88)**	**<0.001**	**2.99 (1.89–4.71)**	**<0.001**	

i. Unadjusted odds ratios (ORs) were estimated using separate univariate (bivariate) logistic regression models, with each explanatory variable entered separately.

ii. Variables with p < 0.20 in the univariate analyses were included in the multivariable logistic regression model to estimate adjusted odds ratios (aORs).

iii. Reference categories are indicated, and bold values denote statistical significance at p < 0.05.

iv. Wald Test P-value.

## Discussion

This facility-based, cross-sectional analytic study estimated the prevalence of self-reported symptoms suggestive of FGS and identified associated risk factors among 695 women of reproductive age attending primary healthcare clinics in the iLembe District of KwaZulu-Natal (KZN), South Africa. By examining FGS-suggestive symptoms and associated sociodemographic and behavioural factors among adult women attending primary healthcare facilities, this study extends the evidence base to a population that has been comparatively underrepresented in FGS research.

The overall prevalence of at least one FGS-suggestive symptom observed in this study was high and broadly consistent with estimates reported from comparable endemic settings in sub-Saharan Africa (SSA) [27]. These findings provide an important baseline for monitoring the effectiveness of future FGS control interventions and public health campaigns in this region. A study conducted in Cameroon reported an overall FGS prevalence of 50.7% by clinical examination among women and girls, with common symptoms such as lower abdominal pain, vaginal itch, and coital pain aligning with symptom-based detection [28]. A retrospective study examining the burden of FGS across endemic countries reported prevalence estimates of 79.5% in Ghana in 2013, 57.5% and 75% in Zambia in 2016 and 2017, respectively, and 61.4% in Tanzania in 2018 [27]. The prevalence of FGS-suggestive symptoms observed in the present study (45.9%) falls within the broad range reported across SSA endemic settings (10.6–75%) [5], including 41.4% in Nigeria [29], 26.9% in Malawi [30], 27.4% (self-reported) in Nigerian endemic communities [31], and 15.8–87% in Tanzania [32], depending on the diagnostic method, with a systematic review confirming this 10.6–75% range across sub-Saharan Africa [5]. However, it is somewhat lower than the estimates reported in some of these studies. These differences may reflect variations in the study design, diagnostic approaches, and population characteristics. While several studies have relied on clinical examination, colposcopy, and/or molecular diagnostics to confirm FGS [28–30,32–34], the present study employed a symptom-based approach, which may capture a broader but less specific spectrum of genital morbidity. In addition, our study population consisted of women of reproductive age who attended primary healthcare facilities, except for those aged 15–17 years, for ethical and legal reasons. In contrast, many previous studies focused on school-aged girls or community-based samples in high-transmission areas [20,23,28,35,36]. Despite these differences, symptom-based assessments provide valuable complementary insights into the potential burden of FGS in routine healthcare settings. For example, FGS studies in Ogun State, Nigeria, used symptom-based assessments to develop an FGS training guide for health workers, enabling primary health care providers to diagnose and manage FGS cases in 22 health facilities [37]. They may support broader surveillance and early case identification in resource-limited contexts where diagnostic tools are not widely available.

The predominance of lower abdominal pain (40.8%), vaginal itching (34.5%), and abnormal vaginal discharge (27.8%) in this study mirrors the patterns documented in clinical and community-based FGS studies from Tanzania [38] and Zimbabwe [34]. Self-reported symptoms in Nigeria showed high rates, such as genital itching (48.7%) and vaginal discharge (48.2%), in nearly half of the participants from endemic regions [29]. Although our findings are based on self-reported suggestive symptoms rather than clinical diagnoses, these manifestations align with the recognised symptomatology of FGS. Future research could explore the development of differential weighting or symptom-based scoring systems to enhance the diagnostic utility of self-reported FGS symptoms, potentially informed by clinical expertise and existing validated symptom scales. Notably, the co-occurrence analysis in this study revealed that blood in urine combined with lower abdominal pain, and vaginal itching combined with abnormal vaginal discharge, were the most frequent two-symptom combinations among symptomatic women, reflecting symptom clustering consistent with the urogenital pathology of S. haematobium [8,38,39].

Women identifying with Traditional African Religion (TAR) had significantly higher odds of reporting FGS-suggestive symptoms than Christian participants (aOR = 6.37). In this context, religious identity may serve as a proxy for a constellation of related social, behavioural, and environmental factors. Ceremonial and cultural practices associated with TAR, including rituals involving freshwater immersion, bathing in rivers or streams for spiritual cleansing, and the use of communal water bodies, may increase exposure to S. haematobium cercariae. [40,41]. These findings underscore the importance of culturally sensitive public health interventions, including community-based health education developed in collaboration with traditional leaders and practitioners, to promote safer water-contact practices while respecting cultural beliefs.

The results show that women with employer-based income were less likely to report FGS-suggestive symptoms than those relying on remittances. This finding aligns with the established associations between socioeconomic status and infectious disease burden, whereby formal employment confers greater economic security, improved living conditions, better access to piped water, and a greater ability to seek healthcare at the early symptom onset [40]. Conversely, women who depend on remittances are more likely to reside in economically marginalised settings with inadequate water, sanitation, and hygiene (WASH) infrastructure, a greater reliance on natural water bodies for domestic activities, and limited access to healthcare [41]. Targeted socioeconomic support programmes that prioritise improvements in WASH infrastructure in economically marginalised communities may therefore play an important role in reducing FGS risk.

Although most participants reported access to clean running water (81.9%), a much larger proportion (88.8%; n = 617) reported using a natural freshwater source for at least one activity, including bathing, washing clothes, swimming, or other activities. This confirms that continued contact with fresh water is not precluded by greater access to water infrastructure in this setting [20]. In many rural communities in KwaZulu-Natal, rivers and dams still play a role in domestic, recreational, agricultural, and cultural functions, even where piped water is available at home [42,43]. This suggests that access to infrastructure and behavioural exposure should be considered separate constructs in future FGS epidemiological research.

The observed relationship between awareness and symptom reporting likely reflects differences in symptom recognition rather than true differences in infection risk. Women who are knowledgeable about FGS may be more attentive to genital symptoms and more confident in attributing them to Schistosomiasis exposure. This underscores the importance of health education, suggesting that awareness may influence self-reported symptom measures. This interpretation is consistent with models of illness recognition and healthcare-seeking behaviour, which emphasise that accurate disease awareness facilitates symptom attribution and disclosure [44]. Importantly, this finding also indicates that low overall FGS awareness (15.4%) in this population may be causing substantial underreporting of symptoms and underutilisation of schistosomiasis services, suggesting that community education and sensitisation programmes could substantially improve case identification. The low awareness observed likely reflects both the absence of FGS from routine nursing and primary healthcare training curricula in South Africa and its limited inclusion in community health worker guidelines, gaps that reduce the likelihood of symptom prompting and appropriate referral at the point of care [45,46]. Targeted strategies to address this include integrating FGS recognition and referral pathways into the community health worker training modules delivered under the Ward-Based Outreach Teams (WBOT) programme in KwaZulu-Natal [46,47]. Additionally, media channels, which were the primary source of general health information for 65.6% of participants, represent an underutilised platform for community-level FGS health literacy campaigns that could improve symptom recognition and care-seeking in endemic communities [48,49].

Operationalising FGS symptom screening within existing primary healthcare infrastructure in KwaZulu-Natal is both feasible and necessary. Community health workers and professional nurses at primary healthcare clinics could administer a brief structured symptom checklist covering lower abdominal pain, vaginal itching, abnormal vaginal discharge, haematuria, dyspareunia, and postcoital bleeding during routine reproductive health consultations for women of reproductive age in endemic subdistricts. Women screening positive for one or more symptoms could be referred for further clinical assessment and management according to local protocols and World Health Organization recommendations for schistosomiasis control in endemic areas [50]. This model is consistent with symptom-based screening approaches used for tuberculosis and cervical cancer in South African primary healthcare, where standardised screening tools facilitate case finding and referral [51,52]. Embedding the approach within existing digital health systems, including DHIS2-linked platforms in KwaZulu-Natal, could enable scalable implementation, longitudinal patient tracking, and district-level programme monitoring.

The primary sources of general health information among the participants were the media (65.6%) and healthcare workers (31.5%), with fewer participants reporting contributions from family, friends, or traditional healers. Although these sources were not specific to FGS awareness, they provide insight into the communication channels through which this population most commonly accesses health information. This may also reflect limited awareness and training on FGS among frontline healthcare providers, as the condition is not consistently included in nursing and primary healthcare training curricula in many endemic settings [14]. Consequently, opportunities to provide accurate information, recognise symptoms, and facilitate early referrals may be missed [14]. Strengthening the capacity of primary healthcare providers through standardised FGS training modules focusing on symptom recognition, diagnostic pathways, and patient communication could improve the delivery of accurate information and facilitate the earlier identification of FGS [53].

### Strengths and limitations

This study has several strengths. The relatively large, facility-based sample recruited across eight primary healthcare clinics in two municipalities using proportional allocation enhances representativeness within the study setting. The use of a structured, interviewer-administered questionnaire implemented via REDCap, with real-time data capture, supported data quality and integrity. In addition, the symptom questionnaire was contextually adapted to capture locally relevant manifestations and perceptions of FGS-suggestive symptoms among women of reproductive age, allowing the study to systematically document symptom patterns that may not be captured using generic reproductive health survey tools. This approach provided a practical framework for assessing FGS-related symptom burden in routine primary healthcare settings where diagnostic tools are limited. The multivariate analytical approach, with backward selection and robust Wald tests, enabled the identification of independent predictors while controlling for confounders.

This study has several limitations. The cross-sectional design precludes causal inference, and the temporal relationship between exposure and outcome cannot be established. Symptoms were entirely self-reported without clinical or laboratory confirmation, and the outcome measure therefore captured FGS-suggestive symptoms rather than confirmed FGS morbidity. Several reported symptoms, including vaginal itching, abnormal vaginal discharge, and lower abdominal pain, overlap with those of other gynaecological conditions and STIs, independent of schistosomiasis exposure. Given that most participants were unmarried but sexually active, STI-related symptom misattribution cannot be excluded and may have contributed to the overestimation of FGS-attributable morbidity. This limitation reflects a broader challenge in FGS research: accessible, validated diagnostic tools for large-scale community screening remain limited in resource-constrained settings, and definitive attribution requires colposcopic or molecular confirmation, as mandated by the parent DUALSAVE-FGS clinical study protocol. Although freshwater contact activities were captured, data on contact frequency, water body type, and proximity to known transmission sites were not collected, limiting the precision with which the individual-level cercarial exposure risk can be quantified. The facility-based sampling approach may introduce health-seeking bias, limiting generalisability to the broader community population. Additional limitations include the exclusion of women aged 15–17 years due to South African minor consent requirements, purposive selection of eight clinics from a larger pool of available facilities, social desirability and recall bias in self-reported data, minor selection bias from the exclusion of 18 participants with incomplete data, and potential seasonal variation in water-contact behaviours and transmission intensity given the defined June–August 2025 collection window.

## Conclusion

This study demonstrated a high prevalence of self-reported symptoms suggestive of FGS among women of reproductive age attending primary healthcare clinics in the iLembe District of KwaZulu-Natal. The identification of religious affiliation with Traditional African Religion, dependence on remittance income, FGS awareness, and bathing as a water-contact activity as significant correlates of symptom reporting provides actionable evidence for program design. These findings reinforce the need to integrate FGS screening, awareness, and treatment into primary healthcare services in endemic communities, along with healthcare worker training, targeted community health education, and attention to structural socioeconomic determinants of schistosomiasis vulnerability. Community health worker guidelines may be strengthened by developing specific training modules for CHWs on FGS symptom identification and referral pathways, thereby building their capacity to act as frontline screeners and improving the professional relevance of their roles in FGS screening. Comprehensive community-based and clinical strategies that address the intersection of poverty, water access, cultural practices, and health literacy are essential to reduce the burden of FGS and its far-reaching reproductive health consequences in this population.

## Supporting information

S1 Table Proportional sample size (PPS) recruited per clinic using the annual headcount (N = 713). (DOCX)

S2 Table Distribution of self-reported FGS-suggestive symptom combinations among symptomatic women (N = 319). (DOCX)

S3 Table Clinic-level outpatient headcount (June–August 2024) confirming the feasibility of PPS-allocated recruitment targets. (DOCX)

S1 Data An anonymised dataset and Stata do-file were used for the analyses presented in this manuscript. (ZIP)

## Abbreviations

aOR: Adjusted Odds Ratio
BREC: Biomedical Research Ethics Committee
CI: Confidence Interval
FGS: Female Genital Schistosomiasis
HIV: Human Immunodeficiency Virus
HPV: Human Papillomavirus
KZN: KwaZulu-Natal
LMIC: Low- and Middle-Income Countries
MDA: Mass Drug Administration
NTD: Neglected Tropical Disease
OR: Odds Ratio
PHC: Primary Health Care
PHREC: Provincial Health Research Ethics Committee
REDCap: Research Electronic Data Capture
SD: Standard Deviation
STI: Sexually Transmitted Infection
TAR: Traditional African Religion
WASH: Water, Sanitation and Hygiene
WHO: World Health Organization
iLembe DMM: iLembe District Municipality
