# Supplementary material for: Prevalence and associated risk factors of self-reported symptoms suggestive of female genital schistosomiasis (FGS) among women in KwaZulu-Natal, South Africa
Source: PLoS Negl Trop Dis. 2026 Aug 26;20(8):e0014633. doi: 10.1371/journal.pntd.0014633 (PMC13514004; doi:10.1371/journal.pntd.0014633)
Supplement: Supplementary file 1 [file pntd.0014633.s001.docx]

**Table S1: Showing the proportional sample size (PPS) recruited per clinic using the annual headcount (N=713).**

| Unique Identification number | Clinic | Headcount | Proportion (%) | Sample Size (PPS) |
| --- | --- | --- | --- | --- |
| NDCL1 | Thafamasi | 11,020 | 5.26 | 38 |
| NDCL2 | Esidumbini | 24,680 | 11.79 | 84 |
| NDCL 3 | KwaNyuswa | 12,204 | 5.83 | 42 |
| NDCL4 | Wosiyane | 14,176 | 6.77 | 48 |
| MDCL1 | Dokodweni | 17,896 | 8.55 | 61 |
| MDCL2 | Isithebe | 84,894 | 40.55 | 289 |
| MDCL3 | Mandeni | 24,935 | 11.91 | 85 |
| MDCL4 | Ohwebede | 19,565 | 9.34 | 66 |
|  | Total | **209,370** | **100** | **713** |

**Note:** Sample sizes were calculated using probability proportional to size (PPS) based on annual outpatient headcount data reported through the KwaZulu-Natal Provincial Department of Health District Health Information System (DHIS). Allocation followed the formula nᵢ = (Nᵢ/N) × n, where nᵢ is the allocated sample size for clinic i, Nᵢ is the annual headcount for clinic i, N is the total headcount across all clinics, and n is the total study sample size (713).
