# Supplementary material for: Prevalence and associated risk factors of self-reported symptoms suggestive of female genital schistosomiasis (FGS) among women in KwaZulu-Natal, South Africa
Source: PLoS Negl Trop Dis. 2026 Aug 26;20(8):e0014633. doi: 10.1371/journal.pntd.0014633 (PMC13514004; doi:10.1371/journal.pntd.0014633)
Supplement: Supplementary file 2 [file pntd.0014633.s002.docx]

**Table S2: Clinic-level outpatient headcount (June–August 2024) confirming the feasibility of PPS-allocated recruitment targets**

| **Clinic** | **Jun 2024** | **Jul 2024** | **Aug 2024** | **3-month total** | **Daily avg** | **PPS target** | **% daily flow needed** |
| --- | --- | --- | --- | --- | --- | --- | --- |
| Thafamasi | 921 | 933 | 880 | 2,734 | 42.1 | 38 | 1.4% |
| Esidumbini | 1,701 | 2,528 | 2,143 | 6,372 | 98.0 | 84 | 1.3% |
| KwaNyuswa | 933 | 1,156 | 1,034 | 3,123 | 48.0 | 42 | 1.3% |
| Wosiyane | 1,221 | 1,431 | 1,338 | 3,990 | 61.4 | 48 | 1.2% |
| Dokodweni | 1,355 | 1,661 | 1,509 | 4,525 | 69.6 | 61 | 1.3% |
| Isithebe | 6,338 | 7,264 | 7,532 | 21,134 | 325.1 | 289 | 1.4% |
| Mandeni | 1,853 | 2,307 | 2,087 | 6,247 | 96.1 | 85 | 1.4% |
| Ohwebede | 1,582 | 1,578 | 1,698 | 4,858 | 74.7 | 66 | 1.3% |

*Source: KwaZulu-Natal Provincial Department of Health District Health Information System (DHIS), headcount data for June–August 2024, the most recent comparable three-month period available prior to data collection. Daily average calculated over 65 working days (13 weeks x 5 days). % daily flow needed = (PPS target / 65) / daily average attendance.*
