# Supplementary material for: Prevalence and associated risk factors of self-reported symptoms suggestive of female genital schistosomiasis (FGS) among women in KwaZulu-Natal, South Africa
Source: PLoS Negl Trop Dis. 2026 Aug 26;20(8):e0014633. doi: 10.1371/journal.pntd.0014633 (PMC13514004; doi:10.1371/journal.pntd.0014633)
Supplement: Supplementary file 3 [file pntd.0014633.s003.docx]

**Table S3: Distribution of self-reported FGS-suggestive symptom combinations among symptomatic women (N = 319)**

| **Number of symptoms reported** | **n** | **%** | **Most common combinations** |
| --- | --- | --- | --- |
| 1 symptom only | 212 | 66.5 | Lower abdominal pain only (LAP): 64 (20.1%) |
|  |  |  | Vaginal itching only (VI): 58 (18.2%) |
|  |  |  | Abnormal vaginal discharge only (AVD): 39 (12.2%) |
|  |  |  | Blood in urine only (BU): 34 (10.7%) |
|  |  |  | Pain during sex only (PSI): 13 (4.1%) |
|  |  |  | Bleeding after sex only (BSI): 4 (1.3%) |
| 2 symptoms | 79 | 24.8 | BU + LAP: 27 (8.5%) |
|  |  |  | VI + AVD: 17 (5.3%) |
|  |  |  | LAP + VI: 7 (2.2%) |
| 3 symptoms | 20 | 6.3 | LAP + VI + AVD: 5 (1.6%) |
|  |  |  | BU + LAP + AVD: 3 (0.9%) |
|  |  |  | BU + LAP + VI: 3 (0.9%) |
| 4 symptoms | 7 | 2.2 | BU + LAP + VI + AVD: 3 (0.9%) |
| 5 symptoms | 1 | 0.3 | LAP + PSI + BSI + VI + AVD: 1 (0.3%) |
| Total | 319 | 100.0 |  |

*Abbreviations: LAP = lower abdominal pain; VI = vaginal itching; AVD = abnormal vaginal discharge; BU = blood in urine; PSI = pain during sexual intercourse; BSI = bleeding after sexual intercourse. Percentages are calculated among the 319 women who reported at least one FGS-suggestive symptom.*
